# A Blurred Vision of Health: Metabolic Syndrome as a Risk Factor for Glaucoma in a Large Taiwanese Population Study

**DOI:** 10.7150/ijms.121641

**Published:** 2026-01-01

**Authors:** Sheng-Hao Chang, Jia-In Lee, Szu-Chia Chen, Shu-Pin Huang, Jiun-Hung Geng

**Affiliations:** 1 Department of Post Baccalaureate Medicine, Kaohsiung Medical University, Kaohsiung, Taiwan.; 2 Department of Psychiatry, Kaohsiung Medical University Hospital, Kaohsiung Medical University, Kaohsiung, Taiwan.; 3 Department of Internal Medicine, Kaohsiung Municipal Siaogang Hospital, Kaohsiung Medical University, Kaohsiung, Taiwan.; 4 Department of Internal Medicine, Division of Nephrology, Kaohsiung Medical University Hospital, Kaohsiung Medical University, Kaohsiung, Taiwan; 5 Faculty of Medicine, College of Medicine, Kaohsiung Medical University, Kaohsiung, Taiwan.; 6 Research Center for Environmental Medicine, Kaohsiung Medical University, Kaohsiung, Taiwan.; 7 Graduate Institute of Clinical Medicine, College of Medicine, Kaohsiung Medical University, Kaohsiung, Taiwan.; 8 Department of Urology, Kaohsiung Medical University Hospital, Kaohsiung Medical University, Kaohsiung, Taiwan.; 9 Department of Urology, Faculty of Post-Baccalaureate Medicine, College of Medicine, Kaohsiung Medical University, Kaohsiung, Taiwan.; 10 Department of Urology, Kaohsiung Municipal Siaogang Hospital, Kaohsiung, Taiwan.

## Abstract

**Background**: Glaucoma is a leading cause of irreversible blindness that is increasingly being linked to systemic metabolic disturbances. Metabolic syndrome (MetS) is a cluster of metabolic conditions that are associated with various ocular diseases, however the relationship between MetS and glaucoma remains insufficiently explored. This study aimed to investigate the associations between MetS and its components of central obesity, impaired glucose tolerance, high blood pressure, high blood triglycerides, and low high-density lipoprotein with glaucoma in a large Taiwanese population.

**Methods**: A cross-sectional analysis was conducted using data from the Taiwan Biobank, encompassing 93,905 participants aged 40 years and older. MetS was defined according to the National Cholesterol Education Program Adult Treatment Panel III (NCEP-ATP III) criteria, and glaucoma status was determined through self-reported diagnoses. Statistical analyses included logistic regression models adjusted for demographic and clinical variables.

**Results**: The prevalence of MetS in the study population was 25.6%, and the association between MetS and glaucoma was significant (adjusted odds ratio [OR] = 1.15; 95% confidence interval [CI]: 1.02-1.30). A dose-response relationship was observed, with the risk of glaucoma increasing along with the number of MetS components, with a peak OR of 1.36 for individuals with four MetS components. Among the MetS components, impaired glucose tolerance showed the strongest association with glaucoma (OR = 1.17; 95% CI: 1.03-1.32). MetS was an independent risk factor for glaucoma, and the risk increased in parallel with the number of metabolic abnormalities.

**Conclusions**: These findings show the importance of metabolic health in preventing glaucoma and suggest that targeted screening and early interventions may help to mitigate the risk of glaucoma in individuals with MetS.

## Introduction

Glaucoma is a leading cause of irreversible blindness worldwide, in which progressive optic neuropathy leads to visual field loss 1. Epidemiological studies have reported an estimated global prevalence of glaucoma of around 3.5% in individuals aged 40 to 80 years, and that the prevalence increases with age 2. In East Asia, glaucoma affects approximately 3.7% of the adult population, with primary open-angle glaucoma (POAG) being the most common subtype 3. The pathogenesis of glaucoma is multifactorial, and involves elevated intraocular pressure (IOP), oxidative stress, vascular dysregulation, and neurodegeneration 4. Risk factors known to be associated with glaucoma include older age, family history, myopia, diabetes mellitus, hypertension, and dyslipidemia 4. If left untreated, glaucoma can lead to significant visual impairment and blindness, substantially affecting patients' quality of life 1. Given these serious consequences, identifying and understanding the risk factors associated with glaucoma is critical to allow for the early detection and prevention. Moreover, glaucoma has increasingly been recognized to have systemic implications beyond ocular health. Recent studies have highlighted potential links between metabolic dysfunction and glaucomatous neurodegeneration, and therefore the roles of systemic inflammation, endothelial dysfunction, and oxidative stress in the progression of glaucoma warrant further investigation 5. Furthermore, as glaucoma progresses silently in its early stages, early detection and appropriate risk stratification are crucial to mitigate its impact on visual function and quality of life 1. Understanding systemic contributors to the pathophysiology of glaucoma may lead to the development of novel therapeutic approaches targeting metabolic health and mitigate the burden of the disease.

Metabolic syndrome (MetS) has been associated with increased risks of cardiovascular disease and type 2 diabetes mellitus, and it is defined as a cluster of metabolic abnormalities including central obesity, insulin resistance, hypertension, dyslipidemia, and hyperglycemia 6. The prevalence of MetS varies by population, however is estimated to affect approximately 20-25% of the global adult population 7. In Taiwan, the prevalence of MetS in adults is approximately 25-30%, with higher prevalence associated with older age and a sedentary lifestyle 8, 9. MetS has been implicated in the pathogenesis of chronic diseases including cardiovascular disease, stroke, chronic kidney disease, and neurodegenerative disorders 6, 10, 11. Emerging evidence suggests that MetS may also be linked to ocular conditions such as diabetic retinopathy, age-related macular degeneration, and cataracts 12. However, the potential association between MetS and glaucoma has seldom been explored and remains unclear.

Several mechanisms have been proposed to explain how MetS could contribute to glaucoma development. Insulin resistance and chronic low-grade inflammation, both key characteristics of MetS, have been linked to impaired vascular autoregulation and increased oxidative stress, which may exacerbate optic nerve damage 13, 14. Hypertension, another major component of MetS, has also been associated with altered ocular perfusion pressure, potentially contributing to glaucomatous optic neuropathy 15. Furthermore, dyslipidemia, another key characteristic of MetS, may also impact the progression of glaucoma by influencing lipid metabolism in retinal ganglion cells 16, 17. Given these potential links, it is essential to investigate whether MetS is an independent risk factor for glaucoma and whether specific components of MetS have a greater influence on the development of glaucoma.

Compared with previous population-based studies 1-3 from South Korea and Singapore, our analysis uses the Taiwan Biobank, one of the largest Asian biorepositories integrating lifestyle and clinical variables, to provide the first large-scale evaluation of the dose-response relationship between the cumulative burden of MetS components and glaucoma risk in an ethnically homogeneous East Asian population. Therefore, we conducted this study of over 90,000 participants from the Taiwan Biobank (TWB) to examine the association between MetS and glaucoma, and further analyze the impact of individual MetS components on glaucoma. This research offers new perspectives on the intersection of ocular health and metabolic disorders, contributing to potential public health screening and intervention strategies and the earlier identification of those at increased risk. Moreover, our results may provide evidence supporting lifestyle modifications and systemic interventions to improve metabolic health, ultimately reducing the burden of glaucoma in high-risk populations.

## Materials and Methods

### Data Source and Study Population

The TWB was established by the Ministry of Health and Welfare with ethical approval granted by both the Ethics and Governance Council and Institutional Review Board on Biomedical Science Research at Academia Sinica. The goal of the TWB is to improve healthcare quality by addressing the aging population and burden of chronic diseases. Medical, genetic and lifestyle information on individuals aged 30 to 70 years who have not previously received a cancer diagnosis is stored in the TWB database and made available to researchers 18, 19. Participants in the TWB provide data through a structured enrollment process after giving informed consent. This process includes in-person interviews, physical assessments, and blood sample collection. Key variables recorded during enrollment include body height and weight, hip and waist circumferences, age, sex, the use of tobacco and alcohol, medical history, and education level. Laboratory variables encompass fasting glucose, hemoglobin, triglycerides, total cholesterol, low- and high-density lipoprotein (LDL/HDL) cholesterol, uric acid, and estimated glomerular filtration rate 20. Blood pressure (BP) measurements were made digitally by a member of staff following standard protocols after avoiding stimulants and exercise for 30 minutes, with the mean of 3 measurements used in the analysis. This study adhered to the ethical standards of the Declaration of Helsinki and was approved by the Institutional Review Board of Kaohsiung Medical University Hospital (KMUHIRB-F(I)-20250172).

**Figure 1:**
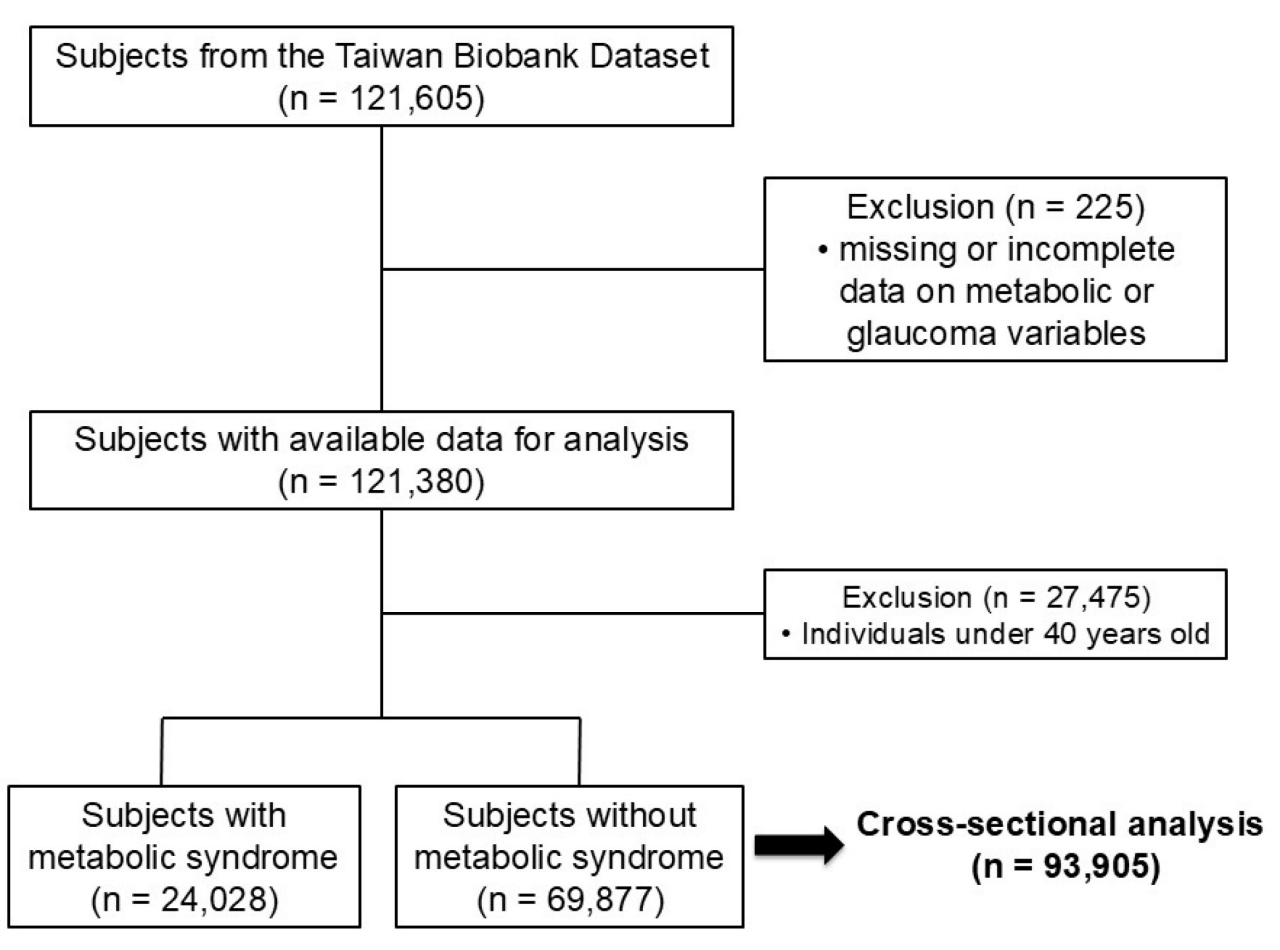
Study participants were classified by Metabolic Syndrome exposure frequency.

There were 121,605 participants in the TWB dataset, and 225 were excluded because of missing or incomplete data on metabolic or glaucoma variables, resulting in 121,380 participants with sufficient baseline data, as shown in **Figure 1**. To specifically examine the role of metabolic factors, individuals younger than 40 years were excluded, as genetic variants following a Mendelian inheritance pattern are the predominant cause of childhood and early-onset glaucoma 21.

### MetS Definition

MetS was diagnosed in individuals who met at least 3 of the following 5 criteria: central obesity (waist circumference of 90/80 cm or higher in men/women), elevated BP (above 130/85 mmHg), high fasting triglycerides (greater than 150 mg/dL), low fasting HDL cholesterol (below 40/50 mg/dL in men/women), and impaired fasting glucose (above 100 mg/dL). Fasting plasma glucose and triglyceride levels were measured after an overnight fast of at least 8 hours. The diagnosis followed the guidelines established by the National Cholesterol Education Program (NCEP) Adult Treatment Panel III (ATP III) 22 and the MetS criteria specific to the Taiwanese population 23.

### Self-reported Glaucoma

The participants' history of glaucoma was obtained during standardized interviews by asking "Have you ever been diagnosed with glaucoma?" Those who stated that they had were classified into the glaucoma group, while the remaining participants were classified into the non-glaucoma group.

### Study Outcome

The aim of this cross-sectional study was to explore the relationship between MetS and the prevalence of glaucoma. In addition to assessing the overall association between these two conditions, we also analyzed separately how the 5 individual components of MetS may contribute to the development or progression of glaucoma.

### Statistical Analysis

The study population was categorized into two groups based on whether or not they were diagnosed with MetS. Categorical variables are summarized as percentages, while continuous variables are summarized as mean ± standard deviation. Differences between continuous and categorical variables were evaluated using the independent t-test and Pearson's χ² test, respectively. To examine the relationship between MetS and glaucoma, univariate and multivariate logistic regression analysis was conducted. The multivariate model was adjusted for variables that showed significant odds ratios in the univariate analysis. Collinearity diagnostics (variance inflation factors, VIFs) were assessed for all covariates, and all values were below 2.0, confirming model stability. Furthermore, sensitivity analyses were conducted to test the robustness of the results, and subgroup analyses were performed to further explore the consistency and potential effect modification of our findings. A p-value below 0.05 was considered significant, and all analyses were performed with SPSS version 20.0 (IBM Corp, Armonk, NY).

## Results

The study cohort consisted of 93,905 participants (33,159 men; 60,746 women; mean age 54±8 years), of whom 24,028 (25.6%) had MetS and 69,877 (74.4%) did not.

### Clinical Characteristics of the MetS Groups

The MetS group were older, had a higher proportion of women and higher body mass index than the non-MetS group (**Table 1**). The MetS group also had higher smoking and alcohol use rates, lower level of education, and were more likely to be married. In addition, they had elevated systolic and diastolic BPs, increased hemoglobin and white blood cell count, and higher prevalence of gout, arthritis, asthma, coronary artery disease, arrhythmia, peptic ulcer, gastroesophageal reflux, cataracts, and chronic kidney disease. Regarding MetS components, an increased waist circumference was the most common, followed by hypertension (**Table 1**).

### Association between MetS and the Risk of Glaucoma

A binary logistic regression analysis without adjustments indicated that the individuals with MetS had a 1.46 times higher likelihood of developing glaucoma (odds ratio [OR] = 1.46; 95% confidence interval [CI] = 1.31-1.62) compared to those without MetS (**Table 2**). To account for potential confounders, a multivariate regression analysis was conducted incorporating variables that showed statistical significance (p < 0.05) in the univariate analysis (**Supplementary Table 1**). These factors were age, sex, education level, systolic BP, hemoglobin A1c, and medical conditions including gout, depression, osteoporosis, arthritis, asthma, emphysema bronchitis, coronary artery disease, arrhythmia, gastroesophageal reflux, irritable bowel disease, cataracts, retinal detachment, floaters and chronic kidney disease.

We also verified that all variables included in the multivariate model had VIF values well below the commonly accepted threshold of 10, indicating no evidence of multicollinearity. The VIF values ranged from 1.010 (asthma) to 1.383 (age), suggesting that all predictors were sufficiently independent and that the regression estimates were stable and reliable (**Supplementary Table 2**). After adjusting for these variables, the association between MetS and glaucoma remained significant, however the OR decreased to 1.15 (OR = 1.15; 95% CI = 1.02-1.30) (**Table 2**). Finally, sensitivity analyses excluding individuals with self-reported ocular comorbidities (retinal detachment and floaters) yielded similar estimates, supporting the robustness of the findings (**Supplementary Table 3**).

### Dose-response Relationship between MetS and the Risk of Glaucoma

There was a dose-response relationship between MetS and glaucoma, and the risk of glaucoma increased along with the number of MetS components. The OR for those with one component was 1.07 (95% CI: 0.91-1.25), and the highest OR was found in those with four components (OR = 1.36; 95% CI = 1.10-1.69). However, a slight decline was noted those with all five components, with the OR decreasing to 1.15 (OR = 1.15; 95% CI = 0.83-1.60) (**Table 2**). A trend analysis treating the number of MetS components as a continuous variable revealed a significant linear association with glaucoma risk (p for trend = 0.007).

### Associations between MetS and its Components with the Risk of Glaucoma

Sub-distribution regression analysis showed significant correlations between the risk of glaucoma and each metabolic component (**Table 3**). Notably, among the five MetS components, impaired glucose tolerance demonstrated the strongest association with glaucoma (OR 1.17; 95% CI = 1.03-1.32) (**Table 3**).

### Subgroup Analyses of the Association Between MetS and the Risk of Glaucoma

To further explore potential effect modification by demographic factors, subgroup analyses were performed stratified by sex and age (**Table 4**). Among men, the adjusted OR for glaucoma associated with MetS was 1.11 (95% CI = 0.92-1.33), whereas among women, the association was slightly stronger (OR = 1.18; 95% CI = 1.01-1.39). When participants were stratified by age group, the association between MetS and glaucoma remained significant in individuals aged 40-64 years (OR = 1.17; 95% CI = 1.02-1.34), but not in those aged ≥ 65 years (OR = 1.13; 95% CI = 0.88-1.44).

## Discussion

The results of this large cross-sectional study of a Taiwanese cohort demonstrated a significant association between MetS and the risk of glaucoma. Individuals with MetS had higher odds of developing glaucoma even after adjusting for potential confounders, indicating that the relationship could not be explained solely by shared risk factors. Notably, a dose-response trend was observed, with the likelihood of glaucoma increasing alongside the number of MetS components. Each component of MetS was positively associated with glaucoma risk, with impaired glucose tolerance emerging as the strongest individual predictor.

Our findings align with several studies from Western populations, although there were some differences. For example, an analysis of over 2 million adults in a U.S. managed-care network found that having diabetes mellitus or hypertension was associated with a higher hazard of developing OAG (hazard ratios ~1.35 and 1.17, respectively), whereas hyperlipidemia alone was associated with a slightly lower risk of glaucoma 24. Similarly, the Blue Mountains Eye Study in Australia reported that the risk of glaucoma was approximately two times higher in individuals with diabetes compared to those without (5.5% vs 2.8%, adjusted OR ~2.12)​, reinforcing the role of glycemic control in reducing the risk of glaucoma 25. On the other hand, a population-based study in the Midwestern United States (Olmsted County) reported that MetS was linked to a higher IOP and greater likelihood of ocular hypertension in glaucoma patients​. However, after accounting for the confounding effect of corneal thickness, the direct association between MetS and glaucoma in that study was attenuated 26. These findings suggest that while metabolic abnormalities (especially impaired glucose tolerance and high BP) increase the risk of glaucoma in both Western and Taiwanese populations, there may be differences in how dyslipidemia or other factors affect the association, possibly due to variations in treatment (e.g. use of lipid-lowering therapy) or population risk profiles. Overall, our results corroborate the general trend observed in Western studies that systemic metabolic abnormalities increase the risk of glaucoma, while also highlighting the complex interplay of individual MetS components 24, 25.

In subgroup analyses, we further examined whether the association between MetS and glaucoma differed by sex or age. The relationship appeared slightly stronger among women than men and was significant among participants younger than 65 years but not in those aged ≥65 years. These findings suggest that metabolic and hormonal factors may play a more prominent role in glaucoma susceptibility among women and younger individuals. Previous studies have also reported similar patterns 25-30. The Korean National Health and Nutrition Examination Survey (KNHANES) found a stronger MetS-glaucoma association in women, possibly reflecting postmenopausal hormonal changes and vascular reactivity differences 30. Likewise, the Singapore Epidemiology of Eye Diseases Study and the Blue Mountains Eye Study observed that metabolic and vascular dysfunction contributed more substantially to glaucoma risk in younger or middle-aged adults than in older individuals, likely due to cumulative vascular adaptation or treatment effects 25, 29. Collectively, these findings support our results and suggest that endocrine and vascular mechanisms may differentially influence glaucoma risk across demographic subgroups.

The positive association between MetS and glaucoma observed in our Taiwanese cohort is also consistent with findings from other Asian populations. A recent nationwide study from South Korea reported a higher prevalence of primary open-angle glaucoma (POAG) among individuals with MetS (5.7%) compared to those without (3.5%), corresponding to an unadjusted OR of 1.85. Even after multivariable adjustment, MetS remained an independent risk factor for glaucoma (adjusted OR ≈ 1.47)​ 27. Other Asian studies have also reported similar trends; for example, an Iranian study found that glaucoma patients had a higher prevalence of MetS components compared to controls​, reinforcing the broad relevance of metabolic health in glaucoma across different Asian ethnicities 28. Overall, the agreement between our results and those from other Asian regions suggests that the association between MetS and glaucoma is not confined to Western populations and may be a global phenomenon.

While most evidence supports a link between MetS and glaucoma, a few studies have reported contradictory findings, and understanding these discrepancies is important. Notably, the Singapore Malay Eye Study found an unexpected inverse relationship, and the participants with MetS had a lower prevalence of glaucoma compared to those without MetS​ 29. This difference may partly be due to variations in study design or population characteristics; for example, the Singapore study was a smaller cross-sectional sample and included relatively few patients with glaucoma, possibly affecting the statistical power or introducing selection bias. In addition, other research has shown no significant association when considering MetS as a whole. For example, a Korean study observed a higher crude prevalence of normal-tension glaucoma in subjects with MetS (2.1% vs 1.5%), but this difference did not reach statistical significance after adjustments (p = 0.067). However, significant associations were found in analysis of individual components, with high BP and impaired glucose tolerance being associated with a higher risk of normal-tension glaucoma 30. These findings imply that examining MetS as a whole may mask the effects of particular components, especially in studies with a limited sample size or specific glaucoma subtypes. Methodological differences may also have contributed to the conflicting outcomes, as some studies relied on self-reported glaucoma or screening IOP measurements, which can misclassify disease status, while others used comprehensive ophthalmologic examinations. Furthermore, variations in how MetS is defined (e.g., different cut-off values for central obesity in Asian and Western criteria) and the degree of control for confounding factors (such as age, medications, or socio-economic status) can lead to heterogeneity in results​ 31. For example, one U.S. study reported that hyperlipidemia was associated with a modestly lower risk of glaucoma, possibly due to the protective effects of long-term statin use or higher HDL cholesterol in those patients ​ 24. Consequently, if a population has a high prevalence of treated dyslipidemia, the net impact of MetS on glaucoma may be blunted or even appear protective, depending on which component is dominant in that population. In summary, divergent findings in the literature likely stem from differences in study population, sample size, interactions between MetS components, and study methodology. Recognizing these inconsistencies highlights the need for caution when interpreting such results, and indicates that a one-size-fits-all explanation may be inadequate - careful consideration of the characteristics of the population and confounders is essential when comparing studies.

Our study provides evidence of a dose-response relationship between MetS and glaucoma, with the risk of glaucoma increasing incrementally with each additional MetS component. This cumulative association has important implications for understanding causality, as it suggests that the cumulative burden of metabolic abnormalities may exert additive strain on the eye. In our data, individuals defined as having MetS (e.g., three or more components) had the highest risk of glaucoma, whereas those with only one or two components had intermediate risk levels compared to metabolically healthy individuals. This pattern of increasing risk with a greater number of MetS components is consistent with findings in other cohorts. For example, a South Korean health survey analysis showed that the prevalence of POAG increased steadily along with the number of MetS components 32​. Similarly, Kim et al. (2014) reported that each additional MetS component conferred about a 10% increase in the odds of normal-tension glaucoma (OR ~1.10 per component) 30. The biological plausibility of this dose-response lies in the additive impact of multiple metabolic derangements: when an individual has several co-existing metabolic issues, the combined effect on vascular health, oxidative stress load, and ocular perfusion may be substantially greater than any single factor alone. This cumulative risk underscores the importance of addressing all components of metabolic health. Clinically, our findings suggest that even partial metabolic control (reducing the number of MetS features) may lower the risk of glaucoma, while conversely that patients who continue to develop metabolic risk factors may warrant closer ophthalmic monitoring. The dose-response trend strengthens the argument for a true association, and consequently further research into whether improving MetS components can stepwise reduce the incidence or progression of glaucoma is warranted.

Among the components of MetS, elevated blood glucose had the strongest association with the risk of glaucoma in this study. This observation is supported by a growing body of evidence suggesting that hyperglycemia and insulin resistance are important contributors to glaucomatous disease. A recent meta-analysis of observational studies found that the MetS components hypertension and hyperglycemia were significantly associated with increased glaucoma risk, whereas obesity and dyslipidemia were not 31. Similarly, in the aforementioned Korean normal-tension glaucoma study, an elevated fasting glucose level (or diabetes status) was one of the two MetS features significantly associated with glaucoma (OR ~1.47) 30. The strong influence of blood sugar has been further corroborated by longitudinal data. For example, the Blue Mountains Eye Study demonstrated a more than two-fold higher odds of OAG in people with diabetes compared to those without, and that diabetes was consistently an independent risk factor for glaucoma across diverse populations 25. The underlying reasons why glycemic control is so pivotal may be related to the deleterious effects of chronic hyperglycemia on the microvasculature and neural tissue. High glucose levels can induce oxidative stress, inflammation, and formation of advanced glycation end-products, which cumulatively damage retinal ganglion cells and the optic nerve over time. Diabetic metabolic dysregulation also impairs autoregulation of ocular blood flow, making the optic nerve more vulnerable to ischemic injury. In contrast, we did not find that central obesity per se was as influential after adjusting for the other factors, and dyslipidemia showed a weaker or inconsistent relationship with the risk of glaucoma, potentially due to counteracting influences of lipid-lowering treatments. Thus, while all components of MetS contribute to some extent, maintaining normal glucose metabolism appears to be particularly crucial in mitigating the risk of glaucoma. Our findings reinforce that patients with prediabetes or diabetes should be regarded as a higher-risk group in glaucoma screening, and that optimal glycemic control may confer ocular benefits in addition to systemic health benefits​ 31.

The association between MetS and glaucoma can be explained by several intersecting pathophysiological mechanisms. First, MetS is characterized by chronic low-grade inflammation and oxidative stress, which can damage ocular tissues. Inflammatory mediators and reactive oxygen species resulting from insulin resistance, visceral adiposity, and dyslipidemia may contribute to retinal ganglion cell apoptosis and optic nerve degeneration 31. These processes can exacerbate glaucomatous neurodegeneration by weakening the supportive glial cells and impairing axonal transport in the optic nerve. Second, vascular dysregulation is a key factor, as long-standing hypertension and atherosclerotic changes associated with MetS can lead to endothelial dysfunction in the ocular microcirculation. This may compromise blood flow to the optic nerve head, thereby reducing perfusion and increasing susceptibility to ischemia, especially in the context of fluctuations in IOP. There is evidence of an association between MetS components with subtle optic nerve structural defects, even in otherwise healthy eyes. For example, localized thinning of the retinal nerve fiber layer has been observed in people with central obesity, high BP, or elevated fasting glucose, even before any overt glaucoma is diagnosed​ 32. Such findings suggest that metabolic stress can initiate early optic neuropathic changes independent of clinically apparent glaucoma. Another mechanism by which MetS may influence glaucoma is through elevation of IOP, the most established risk factor for glaucoma. Systemic hypertension and hyperinsulinemia can increase ciliary body fluid production or alter episcleral venous pressure, leading to higher IOP. Population data have shown that individuals with MetS tend to have higher IOP than their metabolically healthy peers, and that the prevalence of ocular hypertension is greater in individuals with MetS (e.g., 1.3% vs 0.5% in men in one Korean survey) 32. Among the MetS components, hypertension has been identified as a particularly strong predictor of elevated IOP 32, aligning with our finding that BP contributed to the risk of glaucoma. In addition, MetS is often accompanied by structural changes such as increased central corneal thickness (possibly due to insulin-like growth factors or systemic inflammation). Pieńczykowska et al. reported that a thicker cornea could artificially elevate IOP readings and indicate altered extracellular matrix metabolism, although in analysis adjusting for corneal thickness, the difference in IOP between MetS and non-MetS groups diminished​ 32. Beyond IOP, dysregulated glucose and lipid metabolism could directly injure the optic nerve through toxic effects on cells. For example, high glucose can impair astrocyte and microglial function in the optic nerve, while dyslipidemia could contribute to glaucomatous optic nerve head excavation via cholesterol deposition in the trabecular meshwork or blood vessels (although paradoxically, higher HDL might be protective, as noted earlier). The combination of vascular compromise, IOP elevation, and neuroinflammation provides a plausible explanation for why MetS as a whole predisposes individuals to glaucoma 31, 32. It is likely that multiple pathways are involved; for example, a patient with MetS may have modestly higher IOP, reduced optic nerve perfusion and greater oxidative stress, collectively tipping the balance toward the onset or progression of glaucoma. Elucidating these mechanisms further is an active area of research, including studies on mitochondrial dysfunction in diabetic eyes and the neuroprotective potential of anti-inflammatory or insulin-sensitizing therapies in glaucoma. A better understanding of the biological link between MetS and glaucoma could prompt the development of novel preventative strategies such as managing systemic metabolic factors to potentially slow glaucomatous neurodegeneration.

This study has several limitations that should be considered when interpreting the results. First, due to the cross-sectional design we cannot infer causality or the temporal sequence - it is unclear whether MetS predisposes to glaucoma or if, conversely, glaucoma may influence lifestyle factors related to MetS (although the former is more plausible)​ 31. Second, glaucoma was identified based on self-reported questionnaires rather than comprehensive ophthalmologic evaluations. This raises the possibility of misclassification; some glaucoma cases (particularly those asymptomatic or early-stage) may have been missed, while some individuals classified as having glaucoma may not have met the clinical definition. Such non-differential misclassification may have weakened the true association. Third, the definition of MetS was based on single measurements of its components, and we did not have longitudinal data on metabolic status. Metabolic factors can fluctuate or worsen over time, and a one-time assessment may not capture long-term exposure. Likewise, we could not evaluate incident glaucoma after baseline because of the cross-sectional nature of the study. Fourth, unmeasured confounders may have influenced our findings. Although we adjusted for many demographic and health variables, factors such as family history of glaucoma, systemic medication use (e.g., antihypertensives, statins, corticosteroids), and lifestyle factors (diet, physical activity, sleep) were not fully accounted for. For example, the use of statin therapy could potentially have reduced the risk of glaucoma and biased our results towards the null. However, without detailed medication data, such effects are difficult to analyze. Fifth, the study population was ethnically homogeneous (mostly Han Taiwanese) and middle-aged, which may limit the generalizability of our findings to other groups. The association between MetS and glaucoma may differ in other ethnic groups due to differences in genetic risk profiles for glaucoma (for instance, Africans or Europeans) or in younger populations. In addition, our analysis did not differentiate between glaucoma subtypes, limiting our ability to determine whether MetS is more strongly associated with high-tension or normal-tension glaucoma. Given the high prevalence of normal-tension glaucoma in Taiwan 33, metabolic dysregulation may increase optic nerve vulnerability through vascular and neuroinflammatory pathways even at normal IOP levels. Although subtype data were unavailable, this limitation likely attenuates rather than negates the observed association. Another limitation concerns the potential selection bias associated with the Taiwan Biobank dataset. As shown in Table 2, the prevalence of glaucoma was 1.5% among participants without MetS and 2.2% among those with MetS. These estimates are consistent with the prevalence reported in other Asian populations, including Korean (1.7%) and Singaporean (1.9%) cohorts 27-31. However, because the Taiwan Biobank recruits community volunteers rather than a strictly population-based sample, participants may be healthier and more health-conscious than the general population, potentially leading to a slight underestimation of glaucoma prevalence. Finally, survivorship and selection biases are possible: individuals who participate in health screening programs may be more health-conscious or have different health conditions than the general population, and those with very severe metabolic or ocular diseases may not be represented if they were unable to attend the examination. These limitations suggest caution in interpreting the strength of the association and imply that further studies, particularly prospective cohort studies and those with comprehensive glaucoma assessments, are needed to confirm our findings. Despite these limitations, the robust sample size and significant associations observed provide a compelling starting point for deeper investigations into the influences of metabolic factors on glaucoma.

## Conclusion

This study demonstrated a significant association between MetS and an increased risk of glaucoma in a large Taiwanese population. A dose-response relationship was observed, with the risk of glaucoma increasing along with the number of MetS components, particularly impaired glucose tolerance. These findings highlight the importance of metabolic health in glaucoma prevention and suggest that early screening and interventions in individuals with MetS may help reduce disease burden.

## Supplementary Material

Supplementary tables.

## Figures and Tables

**Table 1:** Clinical characteristics of the study participants classified by the presence of metabolic syndrome

Characteristics	Total (N = 93,905)	Metabolic syndrome (N = 24,028)	No metabolic syndrome (N = 69,877)	*p* value
**Demographic data**				
Age, yr	54±8	56±8	54±8	<0.001
Women, n (%)	60,746 (65)	14,040 (58)	46,706 (67)	<0.001
BMI, kg/m^2^	24.3±3.6	27.0±3.6	23.3±3.1	<0.001
Smoke, ever, n (%)	25,486 (27)	7,940 (33)	17,546 (25)	<0.001
Alcohol Status, ever, n (%)	8,474 (9)	2,922 (12)	5,552 (8)	<0.001
Education Status, n (%)				<0.001
< college	46,080 (49)	13,349 (56)	32,731 (47)	
≧ college	47,825 (51)	10,679 (44)	37,146 (53)	
Married, yes, n (%)	87,060 (93)	22,496 (94)	64,564 (92.4)	<0.001
Systolic BP, mm Hg	123±19	134±18	119±18	<0.001
Diastolic BP, mm Hg	75±11	81±11	73±11	<0.001
Hemoglobin A1c, %	5.8±0.8	6.3±1.2	5.7±0.6	<0.001
White Blood Cell, /uL	5.8±1.6	6.2±1.7	5.6±1.5	<0.001
				
**Comorbidities**				
Gout, n (%)	3,984 (4)	1,832 (8)	2,152 (3)	<0.001
Depression, n (%)	3,627 (4)	1,016 (4)	2,611 (4)	0.001
Osteoporosis, n (%)	4,884 (5)	1,242 (5)	3,642 (5)	0.813
Arthritis, n (%)	5,857 (6)	1,888 (8)	3,969 (6)	<0.001
Asthma, n (%)	3,016 (3)	868 (4)	2,148 (3)	<0.001
Emphysema Bronchitis, n (%)	1,203 (1)	342 (1)	861 (1)	0.023
Coronary Artery Disease, n (%)	1,531 (2)	698 (3)	833(1)	<0.001
Arrhythmia, n (%)	4,721 (5)	1,452 (6)	3,269 (5)	<0.001
Peptic Ulcer, n (%)	15,428 (16)	3,953 (17)	11,475 (16)	0.912
Gastroesophageal Reflux, n (%)	14,016 (15)	3,765 (16)	10,251 (15)	<0.001
Irritable Bowel Disease, n (%)	2,339 (3)	580 (2)	1,759 (3)	0.387
Cataract, n (%)	10,833 (12)	3,486 (15)	7,347 (11)	<0.001
Retinal Detachment, n (%)	1,454 (2)	395 (2)	1,059 (2)	0.163
Floaters, n (%)	13,330 (14)	3,381 (14)	9,949 (14)	0.527
Chronic Kidney Disease, n (%)	1,894 (2)	918 (4)	976 (1)	<0.001
**Components of metabolic syndrome**				
High Blood Pressure*, n (%)	38,474 (41)	18,503 (77)	19,971 (29)	<0.001
Impaired Glucose Tolerance†, n (%)	23,289 (25)	14,251 (59)	9,038 (13)	<0.001
Central Obesity‡, n (%)	45,701 (49)	20,893 (87)	24,808 (36)	<0.001
High Triglyceride§, n (%)	21,136 (23)	15,008 (63)	6,128 (9)	<0.001
Low high-density lipoproteinǁ, n (%)	24,427 (26)	15,362 (64)	9,065 (13)	<0.001

BMI = Body mass index; BP = Blood pressure *Systolic blood pressure greater than 130 mm Hg or diastolic blood pressure greater than 85 mm Hg †Fasting glucose level greater than 100 mg/dL ‡Waist circumference greater than 90 cm in men and greater than 80 cm in women. §Serum triglyceride level greater than 150 mg/dL ǁHigh density lipoprotein cholesterol level less than 40 mg/dL

**Table 2:** Odds Ratios for Glaucoma (n = 93,905)

Variables	No. of glaucoma Cases (%)	Number at Risk	Unadjusted Odds ratio (95% CI)	P-value	Adjusted Odds ratio (95% CI)	P-value
**Metabolic syndrome (yes versus no)**						
No	1,076 (1.5)	69,877	1.00 (Reference)	-	1.00 (Reference)	-
Yes	536 (2.2)	24,028	1.46 (1.31 to 1.62)	<0.001	1.15 (1.02 to 1.30)	0.020
**No. of metabolic syndrome components**						
0	284 (1.3)	22,475	1.00 (Reference)	-	1.00 (Reference)	-
1	400 (1.6)	25,794	1.23 (1.06 to 1.43)	0.008	1.07 (0.91 to 1.25)	0.431
2	392 (1.8)	21,608	1.44 (1.24 to 1.69)	<0.001	1.14 (0.96 to 1.34)	0.136
3	303 (2.1)	14,413	1.68 (1.43 to 1.98)	<0.001	1.22 (1.01 to 1.46)	0.037
4	180 (2.5)	7,297	1.98 (1.64 to 2.39)	<0.001	1.36 (1.10 to 1.69)	0.005
5	53 (2.2)	2,318	1.83 (1.36 to 2.46)	<0.001	1.15 (0.83 to 1.60)	0.398
**Trend per 1-component increase (p for trend)**			1.17 (1.12 to 1.21)	<0.001	1.06 (1.02 to 1.11)	0.007

CI = Confidence interval. The adjusted model included age, sex, education level, systolic blood pressure, hemoglobin A1c, and medical conditions including gout, depression, osteoporosis, arthritis, asthma, emphysema bronchitis, coronary artery disease, arrhythmia, gastroesophageal reflux, irritable bowel disease, cataracts, retinal detachment, floaters and chronic kidney disease.

**Table 3:** Odds Ratios for Glaucoma by individual components of metabolic syndrome (n = 93,905)

Individual component of metabolic syndrome	Unadjusted Odds ratio (95% CI)	P-value	Adjusted Odds ratio (95% CI)	P-value
High Blood Pressure*	1.47 (1.33 to 1.63)	<0.001	1.11 (0.96 to 1.28)	0.087
Impaired Glucose Tolerance†	1.66 (1.50 to 1.84)	<0.001	1.17 (1.03 to 1.32)	0.014
Central Obesity‡	1.19 (1.08 to 1.31)	0.001	1.04 (0.94 to 1.16)	0.451
High Blood Triglyceride§	1.15 (1.03 to 1.29)	0.016	1.05 (0.93 to 1.19)	0.450
Low High-density Lipoproteinǁ	1.08 (0.97 to 1.21)	0.158	1.01 (0.89 to 1.14)	0.900

CI = Confidence interval. *Systolic blood pressure greater than 130 mm Hg or diastolic blood pressure greater than 85 mm Hg †Fasting glucose level greater than 100 mg/dL ‡Waist circumference greater than 90 cm in men and greater than 80 cm in women. §Serum triglyceride level greater than 150 mg/dL ǁHigh density lipoprotein cholesterol level less than 40 mg/dL The adjusted model included age, sex, education level, systolic blood pressure, hemoglobin A1c, and medical conditions including gout, depression, osteoporosis, arthritis, asthma, emphysema bronchitis, coronary artery disease, arrhythmia, gastroesophageal reflux, irritable bowel disease, cataracts, retinal detachment, floaters and chronic kidney disease, as well as the individual components of metabolic syndrome.

**Table 4:** Odds ratios for glaucoma in subgroup analyses (n = 93,905)

Variables	No. of glaucoma Cases (%)	Number at Risk	Adjusted Odds ratio (95% CI)	P-value
Male				
MetS, no	413 (1.8)	23,171	1.00 (Reference)	-
MetS, yes	223 (2.2)	9,988	1.11 (0.92 to 1.33)	0.280
Female				
MetS, no	663 (1.4)	46,706	1.00 (Reference)	-
MetS, yes	313 (2.2)	14,040	1.18 (1.01 to 1.39)	0.035
<65 years old				
MetS, no	863 (1.3)	64,208	1.00 (Reference)	-
MetS, yes	400 (1.9)	20,993	1.17 (1.02 to 1.34)	0.028
≥ 65 years				
MetS, no	213 (3.8)	5,669	1.00 (Reference)	-
MetS, yes	136 (4.5)	3,035	1.13 (0.88 to 1.44)	0.350

CI = Confidence interval; MetS = Metabolic syndrome. The adjusted model included age, sex, education level, systolic blood pressure, hemoglobin A1c, and medical conditions including gout, depression, osteoporosis, arthritis, asthma, emphysema bronchitis, coronary artery disease, arrhythmia, gastroesophageal reflux, irritable bowel disease, cataracts, retinal detachment, floaters and chronic kidney disease.
