# Supplementary material for: A Blurred Vision of Health: Metabolic Syndrome as a Risk Factor for Glaucoma in a Large Taiwanese Population Study
Source: Int J Med Sci. 2026 Jan 1;23(1):216–26. doi: 10.7150/ijms.121641 (PMC12702129; doi:10.7150/ijms.121641)
Supplement: Supplementary file 1 — Supplementary tables. [file ijmsv23p0216s1.pdf]

Supplementary Table 1. Association between variables and glaucoma by univariate binary logistic regression analysis

Variables	Odds ratio (95% confidence interval)	P-value
Age	1.08 (1.07-1.08)	<0.001
Men vs Women	1.20 (1.08-1.33)	<0.001
Body mass index	1.01 (0.99-1.02)	0.263
Smoke	0.95 (0.85-1.06)	0.323
Alcohol Status	0.97 (0.82-1.16)	0.761
Education Status	0.89 (0.83-0.97)	0.005
Married	0.94 (0.78-1.13)	0.530
Systolic blood pressure	1.01 (1.01-1.01)	<0.001
Diastolic blood pressure	1.00 (1.00-1.01)	0.785
Hemoglobin A1c	1.22 (1.18-1.27)	<0.001
White Blood Cell	0.97 (0.94-1.00)	0.055
Gout	1.41 (1.14-1.74)	0.001
Depression	1.78 (1.45-2.17)	<0.001
Osteoporosis	1.96 (1.65-2.31)	<0.001
Arthritis	1.84 (1.57-2.16)	<0.001
Asthma	1.36 (1.06-1.73)	0.014
Emphysema Bronchitis	1.94 (1.41-2.68)	<0.001
Coronary Artery Disease	2.52 (1.95-3.25)	<0.001
Arrhythmia	2.04 (1.73-2.42)	<0.001
Peptic Ulcer	1.58 (1.41-1.78)	<0.001
Gastroesophageal Reflux	1.64 (1.46-1.85)	<0.001
Irritable Bowel Disease	1.69 (1.32-2.17)	<0.001
Cataract	5.57 (5.03-6.16)	<0.001
Retinal Detachment	3.12 (2.46-3.97)	<0.001
Floaters	2.51 (2.25-2.80)	<0.001
Chronic Kidney Disease	1.74 (1.32-2.28)	<0.001
Metabolic syndrome	1.46 (1.31-1.62)	<0.001

Supplementary Table 2. Collinearity Diagnostics (Variance Inflation Factor Values) for Variables in the Multivariate Model

Variable	Variance Inflation Factor (VIF)
Age	1.383
Sex	1.170
Education level	1.122
Systolic blood pressure	1.289
HbA1c	1.157
Gout	1.081
Depression	1.015
Osteoporosis	1.054
Arthritis	1.049
Asthma	1.010
Emphysema/Bronchitis	1.013
Coronary Artery Disease	1.025
Arrhythmia	1.019
Gastroesophageal Reflux	1.033
Irritable Bowel Syndrome	1.017
Cataract	1.159
Retinal Detachment	1.022
Floaters	1.068
Chronic Kidney Disease	1.035
Metabolic syndrome	1.261

Supplementary Table 3. Sensitivity analyses of the association between metabolic syndrome and glaucoma

Variables	Adjusted odds ratio (95% CI)	P-value
Exclude participants with retinal detachment		
MetS, no	1.00 (Reference)	-
MetS, yes	1.15 (1.02 to 1.30)	0.027
Exclude participants with floaters		
MetS, no	1.00 (Reference)	-
MetS, yes	1.17 (1.02 to 1.35)	0.030

CI = Confidence interval; MetS = Metabolic syndrome.

The adjusted model included age, sex, education level, systolic blood pressure, hemoglobin A1c, and medical conditions including gout, depression, osteoporosis, arthritis, asthma, emphysema bronchitis, coronary artery disease, arrhythmia, gastroesophageal reflux, irritable bowel disease, cataracts, retinal detachment, floaters and chronic kidney disease.
